# Volume alterations of brainstem subregions in migraine with aura

**DOI:** 10.1016/j.nicl.2019.101714

**Published:** 2019-02-04

**Authors:** Igor Petrusic, Marko Dakovic, Jasna Zidverc-Trajkovic

**Affiliations:** a Laboratory for advanced analysis of neuroimages, Faculty of Physical Chemistry, University of Belgrade, Serbia; b Faculty of Medicine, University of Belgrade, Serbia; c Center for Headaches, Neurology Clinic, Clinical Center of Serbia, Serbia

**Keywords:** Migraine with aura, Brainstem, Midbrain, Pons

## Abstract

**Background:**

The brainstem plays a significant role in migraine pathogenesis, but a relationship between volume alterations of brainstem subregions and migraine aura characteristics has not been sufficiently investigated. The aim of this study is to compare the volume of the brainstem, and its subregions, between patients with a migraine with aura (MwA) and healthy controls (HC), and also to correlate characteristics of MwA and the volume of the brainstem subregions.

**Methods:**

Forty-two MwA and 42 HCs, balanced by sex and age, were selected for this study. Total brainstem volume changes as well as volume changes in the pons, medulla, midbrain and the superior cerebellar peduncles were investigated in MwA relative to HCs. In addition, the relationships between brainstem subregions and aura characteristics (aura duration, the frequency of the aura, occurrence of somatosensory and dysphasic aura, duration of a headache, intensity of headache pain and disease duration) were explored in MwA.

**Results:**

MwA patients had a larger brainstem volume relative to HCs (25,941.35 ± 2559.2 mm^3^ vs. 25,179.32 ± 2019.1 mm^3^; p = .008), as well as the midbrain and pons (6155.98 ± 565.7 mm^3^ vs. 5964.22 ± 457.0 mm^3^, p = .002; 15,105.13 ± 1765.5 mm^3^ vs. 14,539.89 ± 1408.4 mm^3^, p = .007, respectively). Total brainstem volume, as well as volumes of brainstem subregions, were not significantly correlated to the MwA characteristics.

**Conclusion:**

The results of this study reveal that a migraine with aura is associated with a larger volume of the brainstem with a particular involvement of the midbrain and pons.

## Highlights

• Migraineurs with aura had larger volume of pons and midbrain relative to controls.
• Medulla did not significantly differ in volume size between migraineurs and controls.
• Volume of brainstem is not linked to disease duration and intensity of headache pain.

## 1 Introduction

Although a migraine with aura (MwA) is a very common disorder in the general population (Global Burden of Disease Study, 2015), the pathophysiology of the migraine aura and its role as a trigger of a headache still puzzle neuroscientists. Also, the origin of the neuronal mechanisms that underlie the primary condition in patients who are susceptible to MwA is still not known (Marciszewski et al., 2018).

One of the proposed theories suggests that inherently dysfunctional brainstem nuclei could constitute a so-called migraine generator (Weiller et al., 1995). Strong connectivity between the midbrain and brain areas relevant for nociceptive and somatosensory processing was demonstrated in patients with frequent migraine attacks (Mainero et al., 2011). Besides the descending pathways from the brainstem to the cortex, the possibility of existence of ascending pathways involved in the initiation of cortical spreading depolarization followed by depression (CSD) is intriguing (Vinogradova, 2015).

The majority of advanced neuroimaging migraine studies investigated cortical changes in migraine patients. There have been only a few studies that analyzed brainstem volume together with characteristics of migraine attacks. Smaller volumes of brainstem subregions were detected in migraine patients with allodynia and lower heat pain thresholds (Chong et al., 2016). Also, grey matter volume decreases in the spinal trigeminal nucleus and dorsomedial pons were noticed (Marciszewski et al., 2018). Larger periaqueductal grey matter volume was measured in patients with episodic migraine compared to healthy subjects (Chen et al., 2017). However, subanalysis of migraine patients with aura was not performed in these reported studies (Chen et al., 2017; Chong et al., 2016; Marciszewski et al., 2018).

The aim of this study is to compare the volume of the brainstem and its subregions, between patients with MwA and healthy controls (HCs), as well as to correlate characteristics of MwA and the volume of brainstem subregions.

## 2 Methods

### 2.1 Participants

This study included 42 MwA patients and the same number of HCs balanced by sex and age. The research protocol of this study was approved by the Institutional Review Boards at the Neurology Clinic and the Special Hospital for Prevention and Treatment of Cerebrovascular Diseases (SHPTCD). Written informed consent was obtained from all subjects prior to study participation. Patients with typical MwA were recruited through the Headache Center at the Neurology Clinic and diagnosed using the International Classification of Headache Disorders criteria (ICHD-3) (IHS, 2018). MwA patients had no history of neurological disorders other than a migraine and were pain-free for at least 72 h prior and 48 h after their scanning appointment. All migraine patients had an episodic MwA for a minimum of 5 years and did not take migraine preventive medication or opiates for pain control. Also, MwA patients had no gross anatomic asymmetry or clear pathology on their previous MRI scans. HCs were voluntarily recruited from clinical staff or their relatives. They were without chronic pain, headache symptoms, history of neurological or metabolic disorders, nor do they have family members who suffered from a migraine. HCs were excluded if they had more than three tension-type headaches per month. All participants were screened and examined by a neurologist (JZT) to ensure that they met inclusion/exclusion criteria. All examined subjects, MwA and HCs, were right-handed. MwA patients were interviewed by a physician (IP) to obtain information about aura characteristics (average duration of the aura, the frequency of the aura per year, occurrence of somatosensory and dysphasic auras, average duration of a headache, intensity of headache pain and disease duration in years). Participants were referred to the SHPTCD, in order to undergo MRI scans (3D T1 and T2W).

### 2.2 MRI data acquisition

MR examinations of participants were performed using a 1.5 T MR scanner with an eight-channel head coil (Signa, General Electric Healthcare, Milwaukee, WI, USA). The imaging protocol consisted of a T2 weighted spin echo (T2W) in the axial plane (Echo time (TE) = 105.8 ms, repetition time (TR) = 5700 ms, flip angle (FA) = 90^o^, 24 slices with 0.47x1x5 mm^2^ voxels, slice thickness = 5 mm, acquisition matrix 512 × 512) and a three-dimensional T1 weighted fast spoiled gradient-echo (T1-3D-FSPGR) series (TE = 3.60 ms, TR = 8.12 ms, FA = 15^o^, 248 continuous slices with 0.47 × 0.47 × 1.4 mm^3^ voxels, slice thickness = 1.4 mm, acquisition matrix 512 × 512, FOV = 256 × 256 mm^2^). T2W images were only used to exclude the presence of brain lesions.

The images were transferred to the PC workstation and converted to the NifTI-1 (Neuroimaging Informatics Technology Initiative) format using the dcm2nii software. Only T1W FSPGR volumetric images were included in the analysis.

Automated segmentation and volume calculation of the entire brainstem, as well as brainstem subregions (medulla, pons, midbrain and superior cerebellar peduncle (SCP)) was done using the FreeSurfer 6.0 version, a freely available automated image analysis software tool with well-established accuracy and reliability for labeling structural anatomy for post-processing neuroimaging data (http://freesurfer.net) (Fischl et al., 2002). The tool utilizes the principles of the active shape and appearance models placed within a Bayesian framework (Iglesias et al., 2015). Voxels were interpolated to 1 × 1 × 1 mm^3^ using FLIRT (FMRIB's Linear Image Registration Tool). Each subject's brain segmentation result was carefully reviewed for errors by a trained analyst (MD). Moreover, the total intracranial volume was computed for each subject using the high-resolution T1W images with the fully automated tool “Structural Image Evaluation, using Normalization, of Atrophy” (SIENAX from FSL 5.0 package) (Smith et al., 2002).

### 2.3 Statistical analysis

Subject demographics and migraine with aura characteristics were reported using descriptive statistics and compared groups using the Independent samples *t*-test or Chi-squared test, as appropriate. Statistical significance levels were set at p < .05.

The volumes of the whole brainstem, as well as brainstem subregions, were exported to the R statistical program. General linear model (GLM) analysis was used to investigate differences in volume of the brainstem and their subregions between the MwA and HCs, controlling for the effect of age, sex and total intracranial volume to avoid spurious results. Although MwA and HCs were balanced by age and sex, these values were used as co-variables because of suggested atypical age-related cortical thinning in MwA relative to HCs (Chong et al., 2014). The results were corrected for multiple comparisons using a Bonferroni correction with a significance level of p < .01 (p < .05/5). Also, the partial correlation test was used to assess correlations between the volume of brainstem subregions and characteristics of a migraine with aura. The results of correlations were corrected for multiple comparisons using a false-discovery rate of the Bonferroni-Holm correction.

## 3 Results

The study included 32 females and 10 males with a migraine with typical aura balanced with the same number of HCs. The main demographic and clinical characteristics of participants included in the study are shown in Table 1.

**Table 1:** Comparison of demographic data of MA (including aura features) and HCs.

Demographic data/characteristics of the aura	MA (n = 42)	HCs (n = 42)	Statistics
Gender - women, %	32 (76)	32 (76)	p = 1.000
Age of patients, x ± sd, years	40.14 ± 10.6	40.14 ± 11.0	p = 1.000
Years lived with migraine, x ± sd, years	19.48 ± 10.0	/	/
The duration of the aura, x ± sd, minutes	38.10 ± 24.7	/	/
Number of migraine with aura per year, x ± sd	8.31 ± 11.7	/	/
Visual symptoms, %	42 (100)	/	/
Somatosensory symptoms, %	24 (57)	/	/
Dysphasic symptoms, %	22 (52)	/	/
Average headache duration, x ± sd, hours	8.83 ± 12.1	/	/
Headache pain intensity (scale 1–10), x ± sd	6.45 ± 2.5	/	/

MwA - migraineurs with aura, HCs - healthy controls.

MwA had significantly larger total brainstem volume and the volumes of the pons and midbrain relative to HCs (Table 2). The medulla and superior cerebellar peduncle (SCP) did not significantly differ in volume size between the MwA and HCs. The multivariate GLM had observed power = 0.866 (p = .008).

**Table 2:** Comparison of the volume of brainstem and its subregions between MwA and HCs.

Brainstem with subregions	MwA (n = 42)	HCs (n = 42)	Statistics
Brainstem (mm^3^)	25,941.35 ± 2559.2	25,179.32 ± 2019.1	p = .008
Medulla (mm^3^)	4420.27 ± 354.7	4412.28 ± 348.1	p = .754
Pons (mm^3^)	15,105.13 ± 1765.5	14,539.89 ± 1408.4	p = .007
SCP (mm^3^)	259.99 ± 47.6	262.93 ± 38.6	p = .914
Midbrain (mm^3^)	6155.98 ± 565.7	5964.22 ± 457.0	p = .002

MwA - migraineurs with aura, HCs - healthy controls.

There were no significant correlations between volumes of the brainstem, as well its subregions, and the duration of the aura, the frequency of the aura, occurrence of somatosensory and dysphasic auras, duration of a headache, intensity of headache pain, and duration of disease. A negative correlation between the occurrence of dysphasia and volume of SCP (Spearman's test = −0.325; p = .036) did not survive the Bonferroni-Holm correction.

## 4 Discussion

The results of this study reveal that MwA is associated with volume changes of brainstem regions. In particular, MwA patients have larger volumes of the midbrain and pons relative to HCs. In MwA subjects, these grey matter volume changes were not influenced by the duration or frequency of the aura, occurrence of somatosensory and dysphasic symptoms, duration or intensity of headache pain, and duration of disease.

Over the past two decades, a number of brain morphometric studies have explored grey matter changes associated with a migraine with aura focusing mainly on the cerebral cortex (DaSilva et al., 2007; Schmidt-Wilcke et al., 2008; Gaist et al., 2018). These studies report increased cortical volume/thickness in the regions involved in the pathophysiology of migraine aura, such as the visual and somatosensory cortex, and reduced volume in areas related to pain, such as the precentral gyrus, cingulate and insular cortices. Also, there are conflicting results regarding the brainstem volume changes (Chong et al., 2016; Chen et al., 2017; Marciszewski et al., 2018). While some found that migraine patients had a smaller midbrain volume (Chong et al., 2016; Marciszewski et al., 2018), others noted a larger volume of periaqueductal grey matter in migraine patients (Chen et al., 2017). However, none of these studies made a distinction between patients who have a migraine with and without aura, which can have a significant impact on the result and conclusions. There is some evidence that patients that have a migraine with aura and those who have only migraine without aura should be investigated separately as two distinctive disorders (Szabó et al., 2018).

Several neuroimaging studies in migraine patients have shown an altered hypothalamic functional connectivity in the dorsal-rostral pontine region (Schulte and May 2016; Moulton et al., 2014). Further, it is known that nociceptive trigeminal activation could activate the *locus ceruleus*, nuclei in the pons, which can be a mechanism through which migraine triggers cause a migraine (Ter Horst et al., 2001). Moreover, experimental models on animals showed that modulation of the *locus ceruleus* influences susceptibility to CSD, which can induce a migraine aura (Goadsby and Duckworth, 1989). We did not investigate the functional changes in the pons, so we cannot discuss a potential link between the pathophysiology of a migraine with aura and the larger volume of the pons. Further investigations, focused on MwA patients, with multimodal magnetic resonance imaging should be used to gather more knowledge about this hypothesis.

The midbrain is involved in linking components of the motor system, such as the cerebellum, basal ganglia, and cerebral hemisphere. Periaqueductal grey matter is an important part of the midbrain which has been linked to headaches in several studies (Cohen and Goadsby, 2004). A recent study of Chen and his colleagues (Chen et al., 2017) demonstrated that migraineurs had the largest periaqueductal grey matter compared with healthy controls and migraineurs suffering from chronic migraines. These results are in accordance with the results of our study and support the hypothesis that periaqueductal grey matter structural change might be the result of a disrupted periaqueductal grey matter network in migraine patients.

Admittedly, this study is limited by the lack of comparison with migraine patients without aura, thus our results could not be interpreted as specific for MwA patients. However the strength of the study is the relatively large number of examined patients and that is confirmed by the significance of the applied multivariate GLM. Thus, the obtained results are accurate for the sample and generalizable to the population level. The answer to the question of causality, more precisely is the brainstem volume alteration the cause or consequence of a migraine could not be resolved by the study. Follow-up of the same cohort could possibly shed light on the topic. The third limitation is the use of a FSPGR (not MR-RAGE) T1 sequence with non-isotropic voxels.

In conclusion, the brainstem volumetric investigation of 42 migraine patients with typical aura, done with the help of a special designed routine in the software package (FreeSurfer 6.0 version) for investigations of brainstem subregions, revealed a larger volume of the brainstem, more accurately of the pons and midbrain, in MwA patients relative to HCs. Future investigations exploring pons and midbrain resting and evoked activity may provide new evidence supporting the hypothesis that those structures can play a role in migraine aura pathophysiology.

## Funding

The authors disclosed receipt of the following financial support for the research, authorship, and/or publication of this article: IP and MD received research grant support from the Ministry of Education and Science, Republic of Serbia [project no. III 41005], and JZT received research grant support from the Ministry of Education and Science, Republic of Serbia [project no. 175022].

## Declaration of conflicting interests

The authors declared the following potential conflicts of interest with respect to the research, authorship, and/or publication of this article: IP, MD and JTZ have no conflict of interest.
